# Assessment of the safety of the cationic arginine-rich peptides (CARPs) poly-arginine-18 (R18 and R18D) in *ex vivo* models of mast cell degranulation and red blood cell hemolysis

**DOI:** 10.1016/j.bbrep.2022.101305

**Published:** 2022-07-01

**Authors:** Adam B. Edwards, Frank L. Mastaglia, Neville W. Knuckey, Kwok-Ho Yip, Bruno Meloni

**Affiliations:** a Perron Institute for Neurological and Translational Sciences, First Floor, RR-Block, QEII Medical Centre, Nedlands, Western Australia, Australia; b Department of Neurosurgery, Sir Charles Gairdner Hospital, First Floor, G-Block, QEII Medical Centre, Nedlands, Western Australia, Australia; c Centre for Neuromuscular and Neurological Disorders, The University of Western Australia, Nedlands, Western Australia, Australia; d Centre for Cancer Biology, The University of South Australia and South Australia Pathology, Adelaide, South Australia, Australia

**Keywords:** Cationic arginine-rich peptides, Polyarginine-18 (R18), TAT-NR2B9c, Protamine, Mast cells degranulation, Hemolysis

## Abstract

Our laboratory focuses on the development of novel neuroprotective cationic peptides, such poly-arginine-18 (R18: 18-mer of l-arginine; net charge +18) and its d-enantiomer R18D in stroke and other brain injuries. In the clinical development of R18/R18D, their cationic property raises potential safety concerns on their non-specific effects to induce mast cell degranulation and hemolysis. To address this, we first utilised primary human cultured mast cells (HCMCs) to examine anaphylactoid effects. We also included as controls, the well-characterised neuroprotective TAT-NR2B9c peptide and the widely used heparin reversal peptide, protamine. Degranulation assay based on β-hexosaminidase release demonstrated that R18 and R18D did not induce significant mast cell degranulation in both untreated (naïve) and IgE-sensitised HCMCs in a dose-response study to a maximum peptide concentration of 16 μM. Similarly, TAT-NR2B9c and protamine did not induce significant mast cell degranulation. To examine hemolytic effects, red blood cells (RBCs), were incubated with the peptides at a concentration range of 1–16 μM in the absence or presence of 2% plasma. Measurement of hemoglobin absorbance revealed that only R18 induced a modest, but significant degree of hemolysis at the 16 μM concentration, and only in the absence of plasma. This study addressed the potential safety concern of the application of the cationic neuroprotective peptides, especially, R18D, on anaphylactoid responses and hemolysis. The findings indicate that R18, R18D, TAT-NR2B9c and protamine are unlikely to induce histamine mediated anaphylactoid reactions or RBC hemolysis when administered intravenously to patients.

## Highlights

• Cationic peptides R18, R18D, TAT-NR2B9c and protamine do not stimulate the degranulation of *ex vivo* untreated (naïve) and IgE-sensitised primary human cultured mast cells (HCMCs).
• R18, R18D, TAT-NR2B9c and protamine do not induce significant hemolysis of human red blood cells (RBCs).
• R18, R18D, TAT-NR2B9c and protamine are unlikely to induce histamine mediated anaphylactoid reactions or hemolysis when administered intravenously to patients.

## 1 Introduction

Cationic arginine-rich peptides (CARPs) represent a novel class of neuroprotective agents [1], which are of considerable significance in light of the lack of clinically effective neurotherapeutics for acute (e.g., stroke, traumatic brain injury, hypoxic-ischemic encephalopathy) or chronic (e.g., Alzheimer's disease, Parkinson's disease and motor neuron disease) neurological disorders. CARPs include poly-arginine peptides (e.g., R18), cationic cell penetrating peptides (CCPPs; e.g., TAT) and CCPPs fused to putative neuroprotective peptides (e.g., TAT-NR2B9c). It also includes the arginine-rich peptide protamine, which was approved by the United States Food and Drug Administration (FDA) as a reversal agent for the anticoagulant heparin in 1939 and as a constituent in some insulin preparations to prolong euglycaemia in 1950. The R18 and R18D peptides are lead neuroprotective molecules in our laboratory and have demonstrated beneficial effects in rat models of ischemic stroke, hypoxic-ischemic encephalopathy and traumatic brain injury [[2], [3], [4], [5], [6], [7], [8], [9], [10], [11]] and in a non-human primate stroke model [12]. Given the potential future application of R18 or R18D as an ischemic stroke neurotherapeutic, it is important to establish their capacity to cause undesirable side effects such as mast cell induced anaphylactoid reactions and red blood cell (RBC) hemolysis when administered to patients.

In addition to possessing intrinsic neuroprotective properties, this class of peptide has anti-inflammatory, analgesic, anti-microbial, and anti-cancer actions, as well as a purported ability to cause mast cell degranulation and histamine release [13]. To this end, a direct interaction of CARPs with the mast cell Mas-related G protein-couple receptor X2 (MRGPRX2) is one possible mechanism whereby the peptides could cause mast cell degranulation and an immunoglobulin independent anaphylactoid reaction.

Therefore, given the potential future clinical application of R18 and R18D as neuroprotective therapeutics and the uncertainty surrounding the capacity of cationic peptides to induce histamine release and anaphylactoid reactions, this study examined the safeness of R18 and R18D on anaphylactoid responses, as well as their capacity to cause RBC hemolysis. Importantly, by using both naïve or IgE-sensitised mast cells, especially those derived from humans, provides the best opportunity to detect the degranulating inducing properties of the four peptides *in vitro*, because both types of cells may be present *in vivo*, as opposed to mast cell lines (e.g., laboratory of allergic disease 2 cells; LAD2 cells) and genetically engineered cells (e.g., Chinese hamster ovary cells; CHO cells) overexpressing MRGPRX2. Hence, the aim of this study was to provide information regarding the capacity of R18 and R18D to induce mast cell degranulation, as well as RBC hemolysis, which are adverse events that could impact the safety of the peptides when used clinically.

## 2 Materials and methods

### 2.1 Peptides

R18, R18D and TAT-NR2B9c (also known as NA-1 or nerinetide) peptides were synthesized by Mimotopes (Melbourne, Australia) purified using high performance liquid chromatography and were subjected to hydrolysis and amino acid liquid chromatography analysis to provide a precise measurement of peptide content (Mimotopes). R18 was prepared in the acetate and hydrochloride salts, R18D in the acetate salt and TAT-NR2B9c in the trifluoroacetate salt. Protamine sulfate (protamine) for injection was purchased from Sanofi-Aventis (Paris, France). Details of the peptides used in this study are summarised in Table 1.

**Table 1:** Peptides used in study.

Peptide (counter ion)	Sequence	Net charge at pH 7	MW	Purity	Company
R18 (acetate)	H-RRRRRRRRRRRRRRRRRR-OH	+18	2,829.4	98%	Mimotopes
R18 (HCl)	H-RRRRRRRRRRRRRRRRRR-OH	+18	2,829.4	99%	Mimotopes
R18D (acetate)	H-rrrrrrrrrrrrrrrrrr-OH	+18	2,829.4	98%	Auspep
Tat-NR2B9c (TFA)	H-GRKKRRQRRRKLSSIESDV-OH	+7	2,518.9	98%	Mimotopes
Protamine (sulfate)	H-PRRRRSSSRPIRRRRPRRASR- RRRRGGRRRR-OH	+20	4235.9	N/A	Sanofi-Aventis

Lower case r = d-arginine. TFA = Trifluoroacetic acid. Protamine supplied as a 10 mg/mL solution. Protamine (salmon) amino acid sequence based on sequence in Genebank data base. MW = Molecular weight. N/A: not available.

### 2.2 Generation of human culture mast cells

Human cultured mast cells (HCMCs) were generated as previously described [14]. Briefly, human buffy coat was diluted with sterile phosphate buffered saline (PBS; pH 7.4) at a ratio of 1:1, layered gently over Histopaque®-1077 (1.7 g/L; Sigma-Aldrich) and after centrifugation (600 g, 30 min), the interface containing mononuclear cells was harvested and the remaining red blood cells were disrupted with hemolytic solution (155 mM NH_4_Cl, 10 mM KHCO_3_, 0.1 mM EDTA 2Na). CD34^+^ progenitor cells were enriched by positive immunomagnetic selection using CD34 MicroBeads and an autoMACS Separator (Miltenyi Biotec) according to the manufacturer's instructions. The isolated CD34^+^ cells were transferred into 12-well plates at a density of 5 x 10^6^ cells/mL in IMDM medium (Life Technologies), 50 μM 2-mercaptoethanol (Life Technologies), 1% penicillin-streptomycin (Life Technologies), 0.1% bovine serum albumin (BSA; Sigma Aldrich), 100 ng/mL recombinant human (rh) stem cell factor (SCF), 50 ng/mL rh IL-6 and 1 ng/mL rh IL-3 (all rh cytokines from Shenandoah Biotechnology) and placed in a CO_2_ incubator (5% CO_2_) at 37 °C. The cytokine-supplemented medium was replaced weekly and rh IL-3 was omitted from the medium after the first 2 weeks of culture, at which time the population contained 96% mast cells as determined by May Grünwald-Giemsa staining and by flow cytometric analysis (trypase^+^; 10 μg/mL; Millipore).

### 2.3 β-Hexosaminidase release assay

Untreated (naïve) or IgE-sensitised (sensitised with 1 μg/mL human myeloma IgE for 16 h) mast cells were washed and resuspend in Tyrode buffer (129 mM NaCl, 8.4 mM glucose, 10 mM HEPES, 5 mM KCl, 1 mM MgCl_2_, 1.4 mM CaCl_2_ and 1% BSA at pH 7.4) before plated on the wells of a 96 well v-shaped bottom plate. Peptide at a 2x concentration (final concentrations ranged from 0.125 to 16 μM) in Tyrode buffer was added to the wells, and wells incubated at 37 °C for 1 h in the CO_2_ incubator. Note: peptide doses were selected based on a 17–32 fold higher concentration than peak plasma levels obtained following the intravenous administration of therapeuctic doses of R18D and TAT-NR2B9c, respectively (see Discusion for details). A mixture of PMA (phorbol 12-myristate 13 acetate, 5 μM final concentration) and calcium ionophore A23187 (1 μM final concentration) or anti-IgE (1 μg/mL, Calbiochem) were used as a positive control for the assay. After 1-h, supernatant was removed, and cell pellets were lysed in Tyrode buffer containing 0.5% Triton X-100. Supernatants and corresponding cell lysates were incubated with the substate N-acetyl-β-d-glucosamide (PNAG) solution in citrated buffer at 37 °C for 1 h followed by addition of glycine buffer. Absorbance was then measured with a microplate reader at 405 nM with reference filter at 620 nm. β-Hexosaminidase release were expressed as a percentage of β-hexosaminidase in supernatant compared to total cellular content of β-hexosaminidase (supernatant + cell lysate).

### 2.4 Blood and plasma collection and RBC isolation

Human blood was collected by a qualified phlebotomist and handled using appropriate safety procedures, and the Perron Institute approved the procedure and institutional oversight. Blood was obtained from a volunteer after informed consent via venepuncture into 10 mL K_2_-EDTA vacuum collection tubes (Greiner Bio One, Austria). Plasma was obtained from whole blood in collection tubes after centrifugation for 5 min at 3500 rpm. Subsequently, RBCs were washed three times in 150 mM NaCl and resuspended in PBS to the original blood volume. Washed RBCs and plasma were used for hemolysis studies on the day of collection.

### 2.5 RBC hemolysis assay

One millilitre of washed resuspended RBCs was added to 49 mL PBS with 2% plasma (v/v) or without plasma. One hundred and 50 μL of the cell suspension (±2% plasma) was added to wells of 96-well round bottom plate. Subsequently, 50 μL of peptide (at 4x concentration in PBS; final concentrations ranged from 1 to 16 μM) was added to the wells. A control to induce 100% RBC cell lysis consisted of wells with 50 μL Triton X-100 (1% final concentration in PBS), whereas the PBS control represented 0% cell lysis. Plates were incubated at 37 °C for 1 h in a CO_2_ incubator, centrifuged at 500 g for 5 min, and 150 μL of supernatant in wells transferred to a 96-well flat bottom plate before measuring hemoglobin levels at 490 nm using a plate reader. For each hemolysis assay, replicates for control wells (PBS; 0% cell lysis and 100% cell lysis) and peptide treated wells consisted of n = 4 replicates. Data for the R18D and TAT-NR2B9c peptides was pooled from two independent studies with n = 8. Raw hemoglobin absorbance values were used for data analysis and graphical representations of the results.

### 2.6 Statistical analysis

β-Hexosaminidase release and hemolysis measurements were analysed by one-way analysis of variance, followed by post-hoc Dunnett's test to uncover differences between the vehicle control and peptide treatment groups (GraphPad Prism; version 9.3.1., GraphPad Software Inc). All data are presented as mean ± standard error of mean (SEM). A value of *P* < 0.05 was considered significant for all data analysis.

## 3 Results

### 3.1 Effects of peptides on untreated (naïve) and IgE-sensitised human mast cells

In order to examine the capacity of R18 and R18D and the control CARPs, TAT-NR2B9c and protamine, to induce mast cell degranulation, naïve and IgE-sensitised human mast cells were exposed to the peptides for 1 h. Mast cell degranulation was assessed by measuring the amount of β-hexosaminidase released into the culture supernatant.

**Fig. 1:**
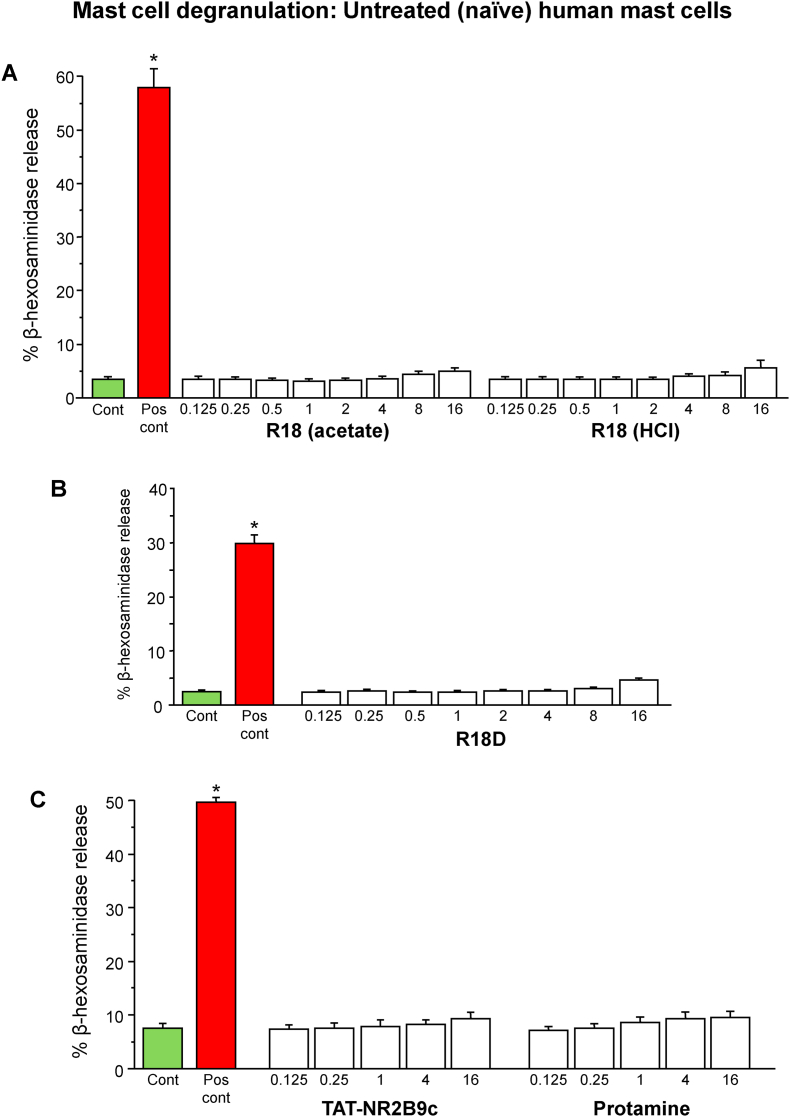
Effects of R18, R18D, TAT-NR2B9c and protamine on naïve mast cell degranulation. (A) R18 acetate and HCl salts, (B) R18D, and (D) TAT-NR2B9c and protamine. The data is presented as mean ± SEM from three independent experiments for each peptide (n = 3). Note: data in graphs A (R18; acetate/HCl), B (R18D) and C (TAT-NR2B9c and protamine) were collected in independent studies. *P < 0.001 when compared with untreated control. Cont = untreated control. Pos cont = positive control; PMA = phorbol 12-myristate 13-acetate (50 ng/mL) and calcium ionophore A23187 (10 μM).

**Fig. 2:**
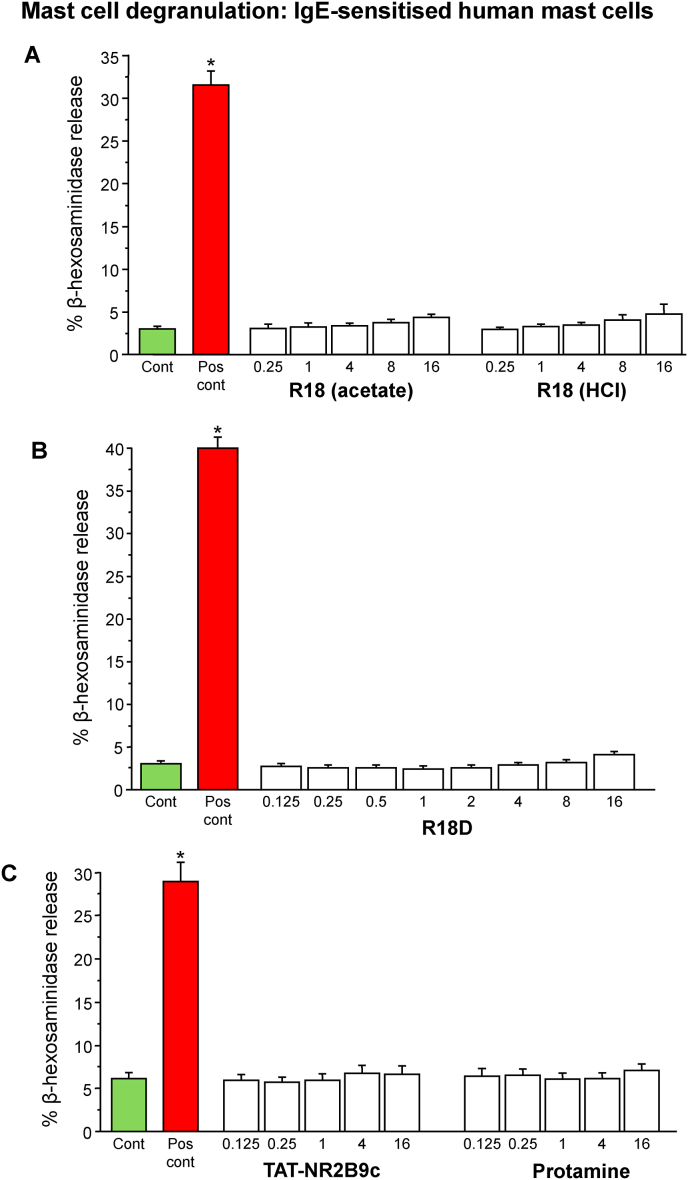
Effects of R18, R18D, TAT-NR2B9c and protamine on IgE-dependent mast cell degranulation. (A) R18 acetate and (B) HCl, (C) R18D, (D) TAT-NR2B9c and (E) protamine. The data is presented as mean ± SEM from three independent experiments for each peptide (n = 3). Note: data in graphs A (R18; acetate/HCl), B (R18D) and C (TAT-NR2B9c and protamine) were collected in independent studies. *P < 0.001 when compared with untreated control. Cont = untreated control. Pos cont = positive control; Anti-IgE at 1 μg/mL.

The exposure of naïve or IgE-sensitised mast cells to R18, R18D, TAT-NR2B9c or protamine peptides for 1 h at concentrations ranging from 0.125 to 16 μM did not induce a significant increase in β-hexosaminidase release compared with the vehicle control (Fig. 1, Fig. 2). A slight increase in β-hexosaminidase supernatant levels of 2–4% above control levels, was observed at the 4–16 μM concentrations, but this is most likely attributable to perturbances of the plasma membrane caused by the cell membrane penetrating actions of the peptides, rather than active mast cell degranulation.

In contrast, the positive controls consisting of PMA/A23187 for naïve cells and anti-IgE for sensitised cells increased β-hexosaminidase release by 27%–53% and 22%–36% above control levels, respectively (*P* < 0.001; Fig. 1, Fig. 2). Taken together, these results indicate that R18, R18D, TAT-NR2B9c and protamine do not stimulate the degranulation of mast cells.

### 3.2 Effects of peptides on human RBC hemolysis

In order to examine the capacity of R18, R18D, TAT-NR2B9c and protamine to induce hemolysis, human RBCs were exposed to the peptides for 1 h. Hemolysis was assessed by measuring hemoglobin released into the culture supernatant.

**Fig. 3:**
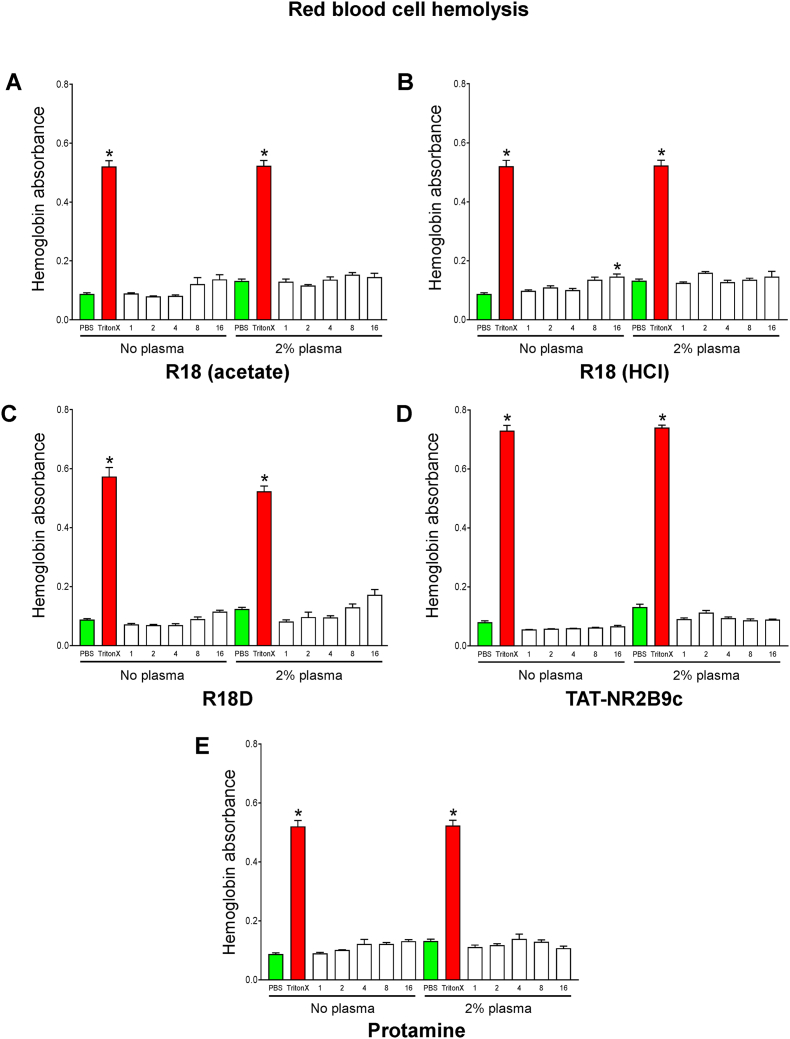
Effects of R18, R18D, TAT-NR2B9c and protamine on red blood cell hemolysis (A) R18 acetate and (B) HCl salts, (C) R18D, (D) TAT-NR2B9c and (E) protamine. The data is presented as mean ± SEM; n = 4 for R18 and protamine and n = 8 for R18D and TAT-NR2B9c. Note: data for R18D and TAT-NR2B9c were collected from two independent studies. *P < 0.001 when compared with untreated control. Triton X-100 (TritonX) control represents 100% RBC lysis. Absorbance 490 nm. (For interpretation of the references to colour in this figure legend, the reader is referred to the Web version of this article.)

The exposure of RBCs to R18 (HCl) without the presence of plasma caused a modest, but statistically significant level of hemolysis at the 16 μM concentration (13.6%; *P* = 0.01), whereas R18D, TAT-NR2B9c and protamine did not cause any significant hemolysis at the concentrations examined (Fig. 3).

The presence of 2% plasma reflects plasma levels in the circulation when peptides are administered intravenously and allows for a more realistic assessment of their impact on RBC lysis. The exposure of RBCs to R18, R18D, TAT-NR2B9c or protamine peptides for 1 h at concentrations ranging from 1 to 16 μM in the presence of 2% plasma did not induce a significant increase hemolysis compared with the vehicle control (Fig. 3). Taken together, these results indicate that R18, R18D, TAT-NR2B9c and protamine do not induce significant hemolysis of RBCs.

## 4 Discussion

Cationic agents such as small molecule drugs, the synthetic polymer 48/80, endogenous substance P peptide, anti-microbial peptides, and wasp venom peptide mastoparan all have the capacity to induce mast cell degranulation and anaphylactoid allergic reactions [[15], [16], [17], [18]]. The reactions range from mild skin itchiness, redness and hives to severe potential life-threatening lowering of blood pressure, increase in heart rate, vasoconstriction, shortness of breath and throat swelling. The binding of cationic agents to the mast cell receptor, Mas-related G protein-coupled receptor X2 (MRGPRX2), is believed to be responsible for the activation and release of histamine and other proinflammatory molecules from mast cell granules that give rise to anaphylactoid symptoms [13].

The findings of the present study indicate that the novel peptides, R18 and R18D, as well as the control peptides, TAT-NR2B9c and protamine, over a wide range of concentrations from 0.125 to 16 μM do not cause any significant degranulation of *ex vivo* derived *in vitro* cultured naïve or IgE-sensitised human mast cells and would therefore be unlikely to induce a mast cell anaphylactoid-mediated reaction if administered to patients. For comparative purposes, it should be mentioned that following intravenous administration of therapeutic doses of R18D (350 nmol/kg) in rats and TAT-NR2B9c (1,032 nmol/kg) in humans, plasma levels peak at 0.9 μM (unpublished observation) and 0.5 μM [19], respectively. Furthermore, no obvious anaphylactoid-like reactions were observed or reported in these studies.

Our findings are in line with recent clinical studies with CARPs, TAT-NR2B9c [19], RD2 (ptlhthnrrrrr-NH_2_; net charge +6.2. Lower case = d-amino acids) [20], CN-105 (Ac-VSRRR-NH_2_; net charge +3) [21] and XG-102/D-JNKI-1 (dqsrpvqpflnlttprkpr-pp-rrrqrrkkrg-NH_2_; net charge +12) [22], which did not uncover any significant peptide related anaphylactoid adverse reactions.

In addition, the polyarginine-9 peptide (R9D/ALX40-4C; Ac-rrrrrrrr-NH_2_; net charge +9) when administered intravenously to human immunodeficiency virus (HIV) positive patients, as a single dose (n = 21) or multiple doses (3 times per week over 4 weeks; n = 40) did not cause any overt allergic reactions. In the multiple dose study, one patient, after the third administration at the highest peptide dose, experienced back pain thought to be caused by an apparent type I allergic reaction, while another patient also treated with the highest dose experienced inflammation of the ocular uvea.

Protamine, when used to reverse heparin anticoagulation, can on rare occasions can cause mild to severe adverse events that range from transient hypotension to bronchospasm and pulmonary hypertension, cardiovascular collapse and death [23]. Diabetics receiving protamine-containing insulin formulations are known to develop an allergy to protamine [24] due to the production of IgE antibodies [25], and are 40 to 50-fold more likely to develop acute adverse events when administered the peptide for heparin reversal [26,27]. Hence, it is likely that severe adverse reactions to protamine are the result of previous exposure to the peptide, causing anaphylaxis (i.e., IgE dependent), as opposed to the molecule causing a direct MRGPRX2-dependent anaphylactoid reaction.

While protamine has been associated with anaphylactoid/anaphylactic adverse reactions these events are relatively rare given its wide clinical usage, and usually occurs in patients previously exposed to the peptide (e.g., diabetics), or who are allergic to fish or have undergone a vasectomy. Therefore, the adverse allergic reactions to protamine are most likely mediated following the production of anti-IgE protamine antibodies which bind the peptide and activate FcεRI receptors on mast cells. The activation of FcεRI receptors elicits a downstream signaling cascade resulting in mast cell degranulation and the release of pro-inflammatory mediators such as histamine [28], prostaglandins and leukotriene C_4_ [29].

Notwithstanding the above, other studies have demonstrated that CARPs have the capacity to stimulate mast cell degranulation and histamine release, apparently via an anaphylactoid (non-IgE-mediated) MRGPRX2 receptor mediated mechanism [30,31]. For example, the TAT-NR2B9c peptide, induced IgE-independent mast cell degranulation *in vitro* at concentrations of 50–50,000 μM [32]. In addition, in a high dose toxicity study TAT-NR2B9c administered intravenously to dogs daily for 14-days at 4,000 nmol/kg (note: clinical dose 1,000 nmol/kg) was accompanied with severe hypotension and elevated plasma levels of histamine immediately after administration (US20190038760A1; [33]). However, given the high TAT-NR2B9c concentrations and doses used in the *in vitro* and *in vivo* studies respectively, one explanation for the positive histamine release findings could be due to the cell penetrating properties of the peptide causing perturbations in mast cell membranes [34] or causing calcium release from intracellular stores [35] resulting in mast cell degranulation. Concomitantly, the protamine peptide, induced significant IgE-independent mast cell degranulation *in vitro* at a concentration of 3,000 μM, but not at lower concentrations (e.g., ≤1,000 μM) [36].

A recent study has examined the capacity of a variety of cationic peptides to activate the human MRGPRX2 receptor stably expressed in CHO–K1 cells, with activation detected by measuring the release of calcium from intracellular stores or receptor association with β-arrestin_2_. Interestingly, all basic peptides examined, including TAT (net charge +8) and polyarginine-9 (R9; net charge +9) caused MRGPRX2 activation; pEC_50_ for TAT and R9 based on calcium mobilisation and B-arrestin_2_ association was 0.63 μM and 5 μM and 0.125 μM and 1.6 μM, respectively. While these findings indicate that cationic peptides bind and activate the MRGPRX2 receptor in CHO–K1 cells stably overexpressing the receptor, the experimental assay may not reflect activation in mast cells, as well as the potential of the peptides to cause anaphylactoid mediated reactions in patients. For example, the CHO–K1 cell activation assay may be more sensitive in detecting MRGPRX2 receptor ligand binding and is not performed in mast cells or in media containing serum or plasma. Therefore, it is important that positive results obtained in assays using genetically modified cells expressing MRGPRX2 be validated *in vitro* using mast cell activation assays and at peptide concentrations that are likely to reflect the *in vivo* situation. Beyond the validation of results in genetically modified cell lines, it has been recently demonstrated that peptide activators of the MRGPRX2 receptor can be reduced to a specific peptide sequence X_a_-(Y)_(n ≥ 3)_-X_b_ where: X_a_ is an aromatic residue; X_b_ is a hydrophobic residue; and Y is a minimum 3 residue long sequence, containing a minimum of one positively charged residue with the remainder being uncharged residues [37].

The R18 peptide induced low level RBC hemolysis and only when exposed to the cells without plasma at highest concentration examined (e.g., 16 μM). The negligible impact of R18, R18D, TAT-NR2B9c and protamine on hemolysis is not surprising given that other studies have generally observed a low capacity of CARPs to induce heamolysis of washed RBCs at physiologically relevant micromolar peptide concentrations [[38], [39], [40]]. In addition, there have been no reports of CARPs that have been used clinically (e.g., protamine, TAT-NR2B9c, CN-105, R9D) of causing hemolysis when administered intravenously to human patients. It is likely that the rapid electrostatic binding of the cationic peptides to plasma proteins and slow intravenous infusion of the peptides would significantly reduce their hemolytic capacity when administered clinically.

While this study has demonstrated that R18, R18D, TAT-NR2B9c and protamine do not elicit IgE-independent degranulation in naïve or IgE-sensitised *in vitro* cultured human mast cells, it does not eliminate the possibility that these cationic peptides may induce allergic reactions, at least in some individuals, as has been demonstrated in the case of protamine. Factors that would increase the likelihood of an allergic reaction include rapid intravenous administration of the peptide resulting in rapid and high tissue concentrations and previous exposure to the peptide. For these reasons peptides such as R18, TAT-NR2B9c and protamine are routinely administrated by slow intravenous infusion over a period of 5–10 min. In addition, while the relatively small size of the peptides reduces the risk of an immunogenic response, this is pertinent when considering the development of therapeutic CARPs for chronic neurological conditions that would require repeated dosing by the oral, subcutaneous or intravenous route. Thus, while R18, R18D, TAT-NR2B9c and protamine are likely to have a low capacity to induce anaphylactoid allergic reactions, further studies are required to determine the capacity of these peptides to induce an anaphylactic response with repeated exposures to the peptide.

In conclusion, this study has demonstrated that the cationic peptides R18, R18D, TAT-NR2B9c and protamine do not stimulate the degranulation of primary HCMCs or induce significant hemolysis of RBCs. Together, the findings indicate that R18, R18D, TAT-NR2B9c and protamine are unlikely to induce histamine mediated anaphylactoid reactions or hemolysis when administered intravenously to patients.

## Funding

The present study was supported by the 10.13039/501100019111Perron Institute for Neurological and Translational Science, and the Department of Neurosurgery at Sir Charles Gairdner Hospital.

## Declaration of competing interest

N.K and B.M are named inventors on several patents for the use of CARPs as therapeutic agents. In addition, N.K, B.M and A.E are shareholders of Argenica Therapeutics, which is a company developing R18 as a stroke therapeutic. D.Y and F.M have no conflicts of interest to declare.
